# Comparative study between staples and eight plate in the management of coronal plane deformities of the knee in skeletally immature children

**DOI:** 10.1007/s11832-016-0758-0

**Published:** 2016-07-14

**Authors:** Arvind Kumar, Sahil Gaba, Alok Sud, Pushpvardhan Mandlecha, Lakshay Goel, Mayur Nayak

**Affiliations:** 1 Department of Orthopaedics, All India Institute of Medical Sciences, New Delhi, India; 2 Department of Orthopaedics, Lady Hardinge Medical College, New Delhi, India; 3 Department of Orthopaedics, Bombay Hospital, Mumbai, India

**Keywords:** Angular deformities around knee, Hemiepiphysiodesis, Staples, Figure of eight plate

## Abstract

**Purpose:**

To compare two commonly used methods of temporary hemiepiphysiodesis (staples and figure of eight plate) in the management of coronal plane deformities of the knee in skeletally immature children.

**Methods:**

This prospective study was conducted between November 2012 and November 2015. A total of 40 patients with 67 affected knee joints, having at least 1 year of skeletal growth remaining, were included in the study. Angular correction was measured by recording the mechanical lateral distal femoral angle (mLDFA), mechanical medial proximal tibial angle (mMPTA), and anatomical tibio-femoral angle (TFA) (for the overall alignment of lower limbs). Implant removal was done after 5° of overcorrection was achieved. The rate of correction (° per month) and complications related to each technique were recorded.

**Results:**

The most common diagnosis was idiopathic genu valgum. The overall rate of correction (TFA) was 1.2° for staples and 1.4° for eight plate (*p* = 0.70, not statistically significant). The correction in mLDFA was statistically better in the eight plate group, whereas an opposite trend was recorded in mMPTA. Implant-related complications were present in two cases of the staples group.

**Conclusion:**

Although the overall correction rate was similar in both groups, implant-related complications were lower with figure of eight plate. In idiopathic genu valgum (the most common diagnosis), the correction was statistically better in the eight plate group. We recommend figure of eight plate over staples in managing these deformities.

## Introduction

Angular deformities around the knee joint are common in the pediatric population. Physiological deformities account for the majority of these cases [1]. While physiological deformities usually correct with growth, pathological deformities can cause functional impairment in the form of abnormal gait, painful joint, and a potential risk of developing osteoarthritis of the knee. Temporary hemiepiphysiodesis, timed permanent hemiepiphysiodesis, corrective osteotomy, and Ilizarov ring fixator application are the various surgical modalities for the correction of angular deformities around the knee joint [2–7]. Corrective osteotomies provide desired correction immediately but are associated with complications like increased blood loss, risk of compartment syndrome, neurovascular injury, and growth disturbance if the growth plate is damaged [2–4]. Prolonged period of immobilization following corrective osteotomy may also cause joint stiffness. Permanent hemiepiphysiodesis has unpredictable results, as the timing of the procedure is never precise. Overcorrection and undercorrection are common complications [7]. Limb length discrepancy can also occur, which may later require correction [7]. Staples, percutaneous screws, or figure of eight plate (tension band plate) can be used for temporary hemiepiphysiodesis [8–10]. It is a less invasive technique compared to osteotomy. The results are more predictable and the process is reversible. The implants can be removed after the desired correction is achieved. Figure of eight plates and epiphyseal staples are both widely used methods of temporary hemiepiphysiodesis [8, 9]. Complications related to the use of staples include breakage, extrusion, and permanent physeal damage [9, 11, 12]. Eight plates have been shown to have fewer complications [9, 13]. Although costlier, figure of eight plates are considered a better alternative to staples because of the fewer complications in comparative studies [14–16]. In this study, we have shared our experience with use of figure of eight plates (Orthofix) and staples (locally manufactured stainless steel staples) for the correction of coronal plane deformities around the knee joint.

## Materials and methods

This prospective study was conducted between November 2012 and November 2015. The included patients had coronal plane angular knee deformities (genu varum and valgum) with at least 1 year of skeletal growth remaining. The following cases were excluded: physiological deformities, deformities due to metabolic disorders that improved with medical management, deformities as a result of infection or neoplasm, post-traumatic deformities, dynamic deformities as in neuromuscular disorders and contractures, non-ambulatory patients.

**Fig. 1:**
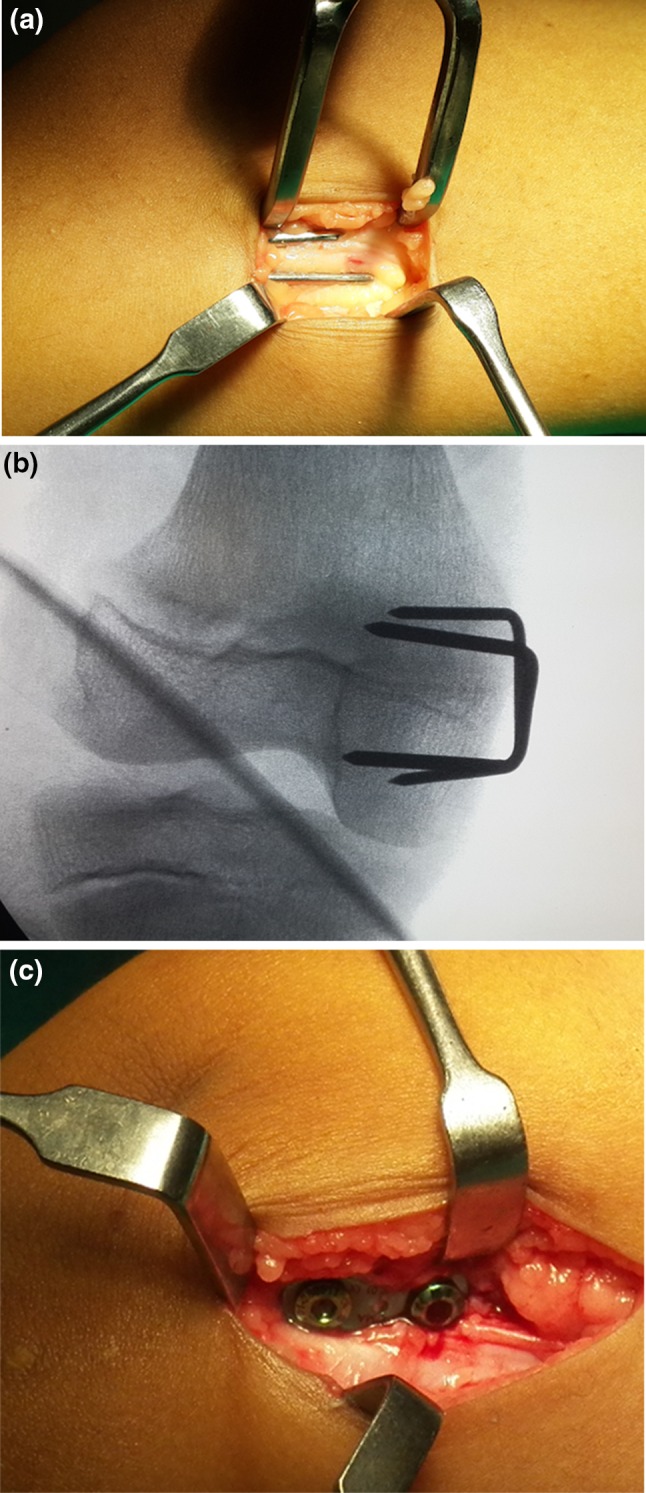
**a** Extraperiosteal placement of staple (intraoperative photograph). **b** Checking correct placement and avoiding physeal penetration under image intensifier. **c** Extraperiosteal placement of eight plate (intraoperative photograph)

The placement of implant in the distal femur, proximal tibia, or both was based on the location of the primary deformity as assessed by abnormality in the mechanical lateral distal femoral angle (mLDFA) and mechanical medial proximal tibial angle (mMPTA). One staple was placed on each side of the midsagittal plane in a submuscular position, with care taken to preserve the periosteum. Intraoperative imaging was used to verify satisfactory hardware placement in both the anteroposterior and lateral planes. Similarly, one eight plate was placed in the midsagittal plane (or slightly posterior to it to avoid future recurvatum deformity), with one screw proximal and one distal to the physis. The entire procedure was extraperiosteal, and care was taken to avoid damage to the physis. Figure 1 depicts intraoperative clinical and radiographic photos. Knee range of motion and full weight-bearing were allowed from the first post-operative day. Crutches or a walker were used to assist in walking in the initial few days. Unassisted weight-bearing was begun as pain subsided. Early return to daily activities was encouraged. The implants were removed after 5° of overcorrection was achieved.

Standing orthoroentgenograms were used to measure the angles. This view allowed assessment of the overall mechanical alignment, mLDFA, mMPTA, and anatomical tibio-femoral angle (TFA). Documentation of these radiological parameters was done at 4-monthly intervals in order to avoid crowding of insignificant observations.

All patients were observed for complications, including wound-related complications, failure to document any improvement after 4 months, implant breakage, implant extrusion, overcorrection (more than 5° in the opposite direction), and physeal penetration. Persistence of deformity 2 years post-surgery was considered as a failure and indication for an alternative procedure in the form of corrective osteotomy.

The end point of correction (time of implant removal) was planned taking into account the “rebound phenomenon”. TFA, mLDFA, and mMPTA were measured, as they are more accurate than clinical examination findings. Aiming for 5° of overcorrection in order to compensate for rebound growth, we kept our end point at 92 ± 3° for mLDFA and 82 ± 3° for mMPTA for valgus deformity correction and 82 ± 3° of mLDFA and 92 ± 3° of mMPTA for varus deformity correction. All patients approaching correction were closely followed every 2 weeks to prevent more than the desired overcorrection. After implant removal, all patients included in the study were closely followed every 2 months to document any recurrence.

SPSS version 20.0 was used for calculation of the mean and standard deviation within the two groups. An independent samples *t* test was used for the comparison of means. A *p*-value of less than 0.05 was considered to be statistically significant.

The institutional review board and ethics committee approved the study. Ethical standards according to the Helsinki declaration of 1964 (and its later amendments) were conformed to. Informed consent was obtained from all patients.

## Results

**Fig. 2:**
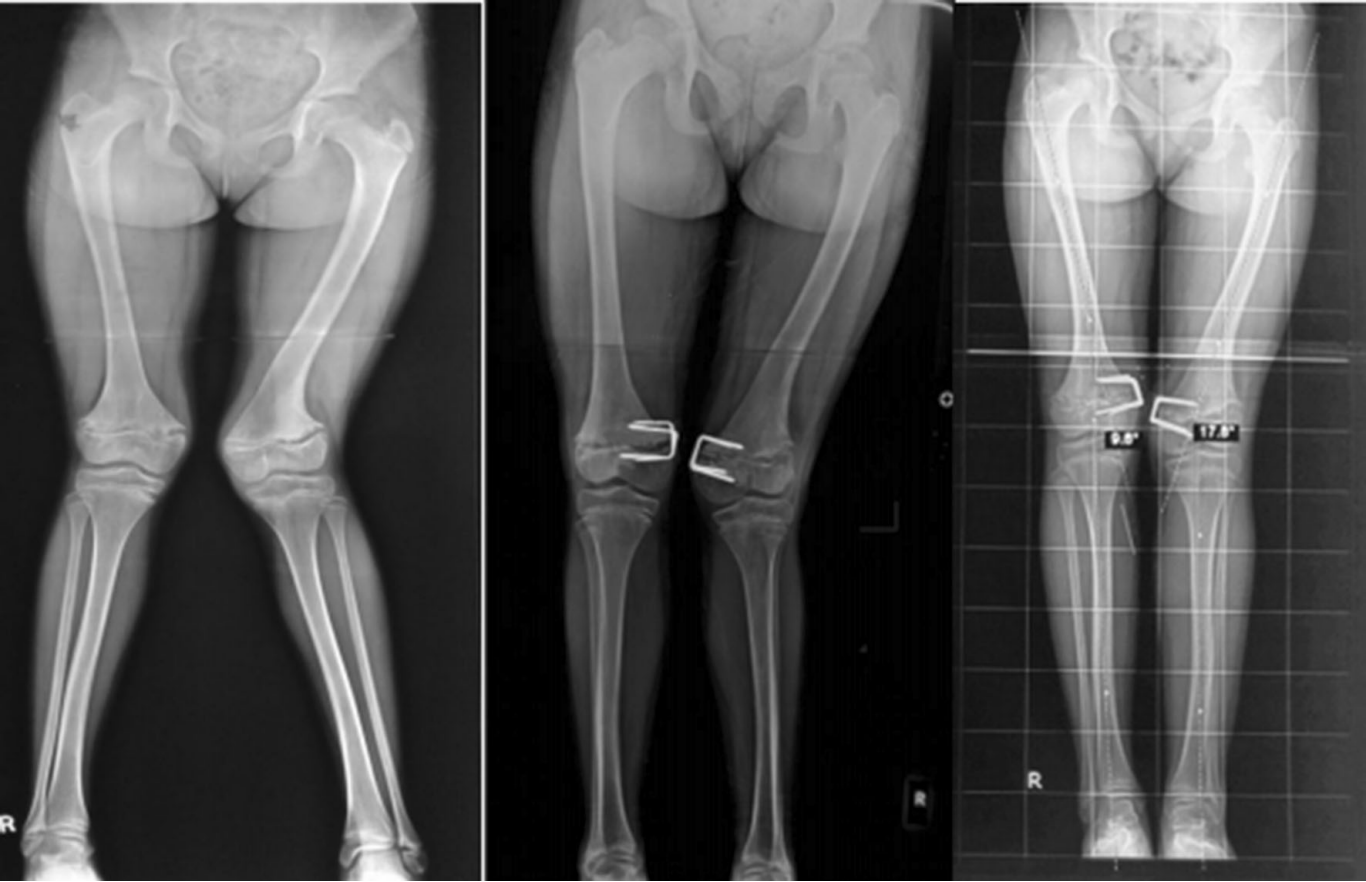
Radiological correction of deformity at 4-monthly intervals with staples

**Fig. 3:**
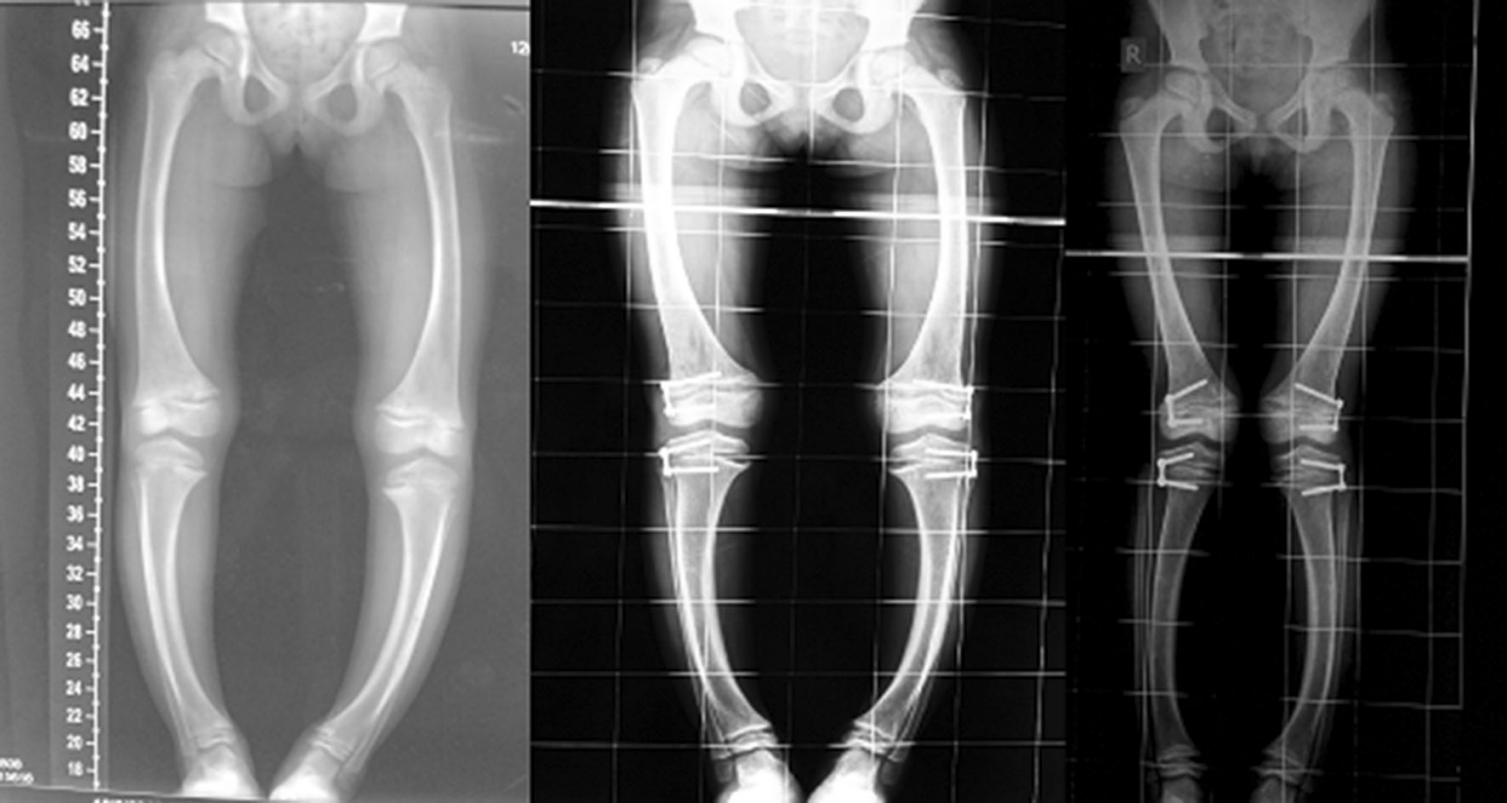
Radiological correction of deformity at 4-monthly intervals with eight plate

Forty patients with 67 affected knee joints were included in the study. Three patients were lost to follow-up. Thirty-seven patients (63 knee joints) with a minimum follow-up of 2 years were available for final assessment. Thirty-one knee joints were included the figure of eight plate group (19 patients), while 32 were included in the epiphyseal staples group (18 patients). Figure 2 shows radiological correction in a case managed with staples and Fig. 3 shows a case managed with eight plate.

**Fig. 4:**
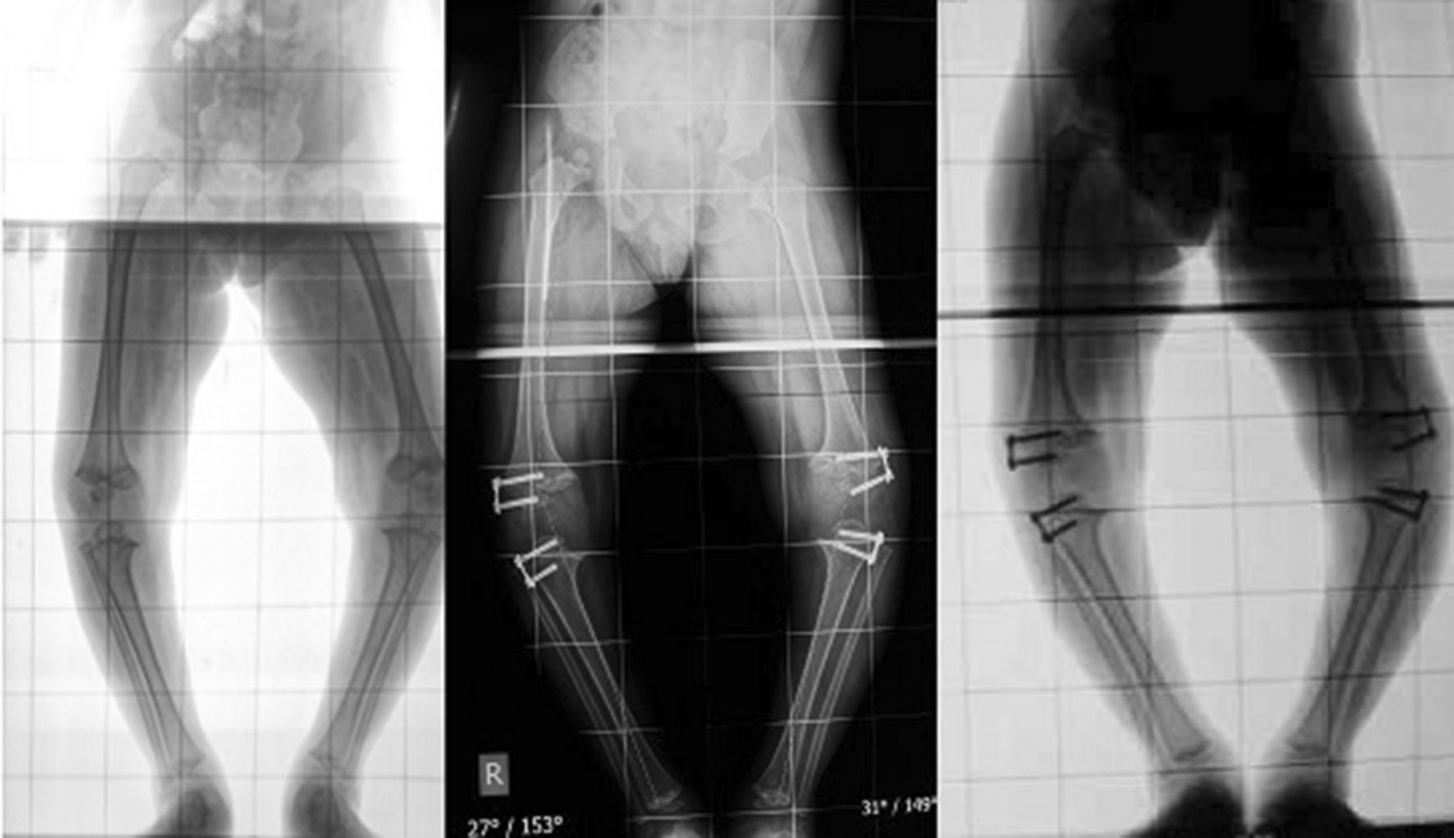
Radiographs showing failure of correction of deformity in a case of skeletal dysplasia managed with eight plate (8 months and 2 years post-operatively)

**Fig. 5:**
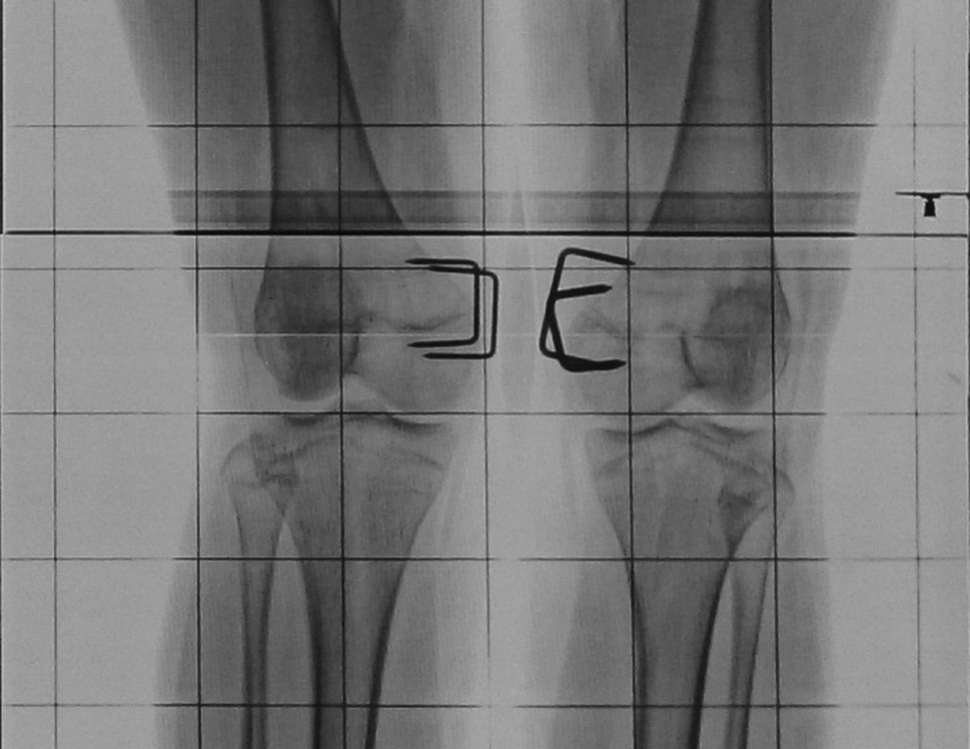
Radiograph showing complication of stapling (migration) 4 months post-operatively

A total of 75 hemiepiphysiodeses were performed on 37 patients (63 in the distal femur, 12 in the proximal tibia). The age and sex distribution, laterality, and number of varus and valgus deformities were similar in both groups (Table 1). The mean age was 7.3 years (range: 3.5–12 years) in the staple group and 7.8 years (range: 4–12.0 years) in eight plate group. The male:female ratio was 8:10 in the staple group and 8:11 in the eight plate group. Idiopathic genu valgum followed by post-rachitic deformity were the most common underlying etiologies. One patient in the eight plate group had bilateral genu valgum deformity after successful treatment of congenital knee dislocation. The mean rate of correction was 1.2° per month in the staples group and 1.4° per month in the eight plate group (Table 2). This difference was not statistically significant (*p* = 0.75). One female patient with bilateral genu valgum managed with staples developed an early complication (implant extrusion) at 4 months and was not considered for calculation of the correction rate. There were no wound-related complications in either group. None of the patients had more than the desired overcorrection at the time of implant removal. A total of four knee joints (two in each group) did not achieve correction by 2 years (Table 3; Fig. 4). The underlying etiology in all these patients was skeletal dysplasia. Thus, an additional procedure was needed in four knee joints (12.5 %) in the staple group (two with failure of correction, two with implant extrusion). Two knee joints (6.5 %) in the eight plate group required additional procedure because of failure of correction. Continuing staples for more than 2 years carries the risk of permanent physeal failure [9]. This complication has not been reported with figure of eight plate. Thus, we consider it wise to remove the implants if deformity persists for more than 2 years. All patients were screened for limb length discrepancy pre-operatively. Pre-operatively, no patient had discrepancy of more than 1 cm. Three patients in the staple group (two unilateral idiopathic genu valgum, one unilateral post rachitic genu valgum) developed shortening of the affected limb of more than 1 cm, while in the eight plate group, two patients with unilateral idiopathic genu valgum developed limb length discrepancy of more than 1 cm. However, this was statistically insignificant. These patients were given appropriate shoe raise and were advised to wait till skeletal maturity for appropriate intervention. A few patients/parents were concerned about implant prominence, and reassurance was all that was required. Staple extrusion occurred in two knees (Fig. 5), while the eight plate group did not show any implant-related complications.

**Table 1:** Demographic profile

Parameters	Staple group	Eight plate group	Remarks
1. Age distribution (years)	Mean = 7.3 (range: 3.5–12)	Mean = 7.8 (range: 4–12)	*p*-value = 0.53 (NS)
2. Sex distribution	Male = 8	Male = 8	*p*-value = 1 (NS)
	Female = 10	Female = 11	
3. Varus/valgus deformity	Varus = 8 knee joints	Varus = 4 knee joints	*p*-value = 0.33 (NS)
	Valgus = 24 knee joints	Valgus = 27 knee joints	
4. Etiology	Skeletal dysplasia = 2	Skeletal dysplasia = 2	
	Idiopathic genu valgum = 20	Idiopathic genu valgum = 18	
	Post-rachitic genu valgum = 4	Post-rachitic genu valgum = 7	
	Post-rachitic genu varum = 6	Post-rachitic genu varum = 2	
		Associated with congenital deformity = 2	
5. Bilateral vs. unilateral	Bilateral = 14 patients	Bilateral = 12 patients	*p*-value = 0.48 (NS)
	Unilateral = 4 patients	Unilateral = 7 patients	
6. Femoral and tibial components involvement	Femur = 32	Femur = 31	*p*-value = 0.36 (NS)
	Tibia = 8	Tibia = 4	

*NS* not statistically significant difference, *SIG* statistically significant difference

**Table 2:** Post-operative evaluation

Parameters	Staple group	Eight plate group	Remarks
Correction rate of mechanical lateral distal femoral angle (° per month)	1.0° per month (SD = 0.35)	1.3° per month (SD = 0.16)	*p*-value = 0.004 (SIG)
	Range: 0.75–1.25	Range: 0.81–2.25	
Correction rate of mechanical medial proximal tibial angle (° per month)	0.9° per month (SD = 0.10)	0.8° per month (SD = 0.29)	*p*-value = 0.001 (SIG)
	Range: 0.75–1.11	Range: 0.56–1.12	
Correction rate of anatomical tibio-femoral angle (° per month)	1.2° per month (SD = 0.37)	1.4° per month (SD = 0.30)	*p*-value = 0.71 (NS)
	Range: 0.75–2.33	Range: 1–2	
Etiology-wise correction rate (° per month)	Skeletal dysplasia = 1.1° (*n* = 2)	Skeletal dysplasia = 1.0° (*n* = 2)	*p*-value = 0.31 (NS)
	Idiopathic genu valgum = 1.0° (*n* = 18)	Idiopathic Genu valgum = 1.3° (*n* = 18)	*p*-value = 0.0001 (SIG)
	Post rachitic genu valgum = 1.1° (*n* = 4)	Post rickets genu valgum = 1.4° (*n* = 7)	*p*-value = 0.14 (NS)
	Post rachitic genu varum = 1.9° (*n* = 6)	Post rachitic genu varum = 1.9° (*n* = 2)	*p*-value = 0.83 (NS)
Time of implant removal	Mean = 12 months	Mean = 10.3 months	*p*-value = 0.54 (NS)

*NS* not statistically significant difference, *SIG* statistically significant difference

**Table 3:** Complications

Complication	Staple group	Eight plate group	Remarks
Wound healing-related complications	NIL	NIL	*p*-value = 1.0 (NS)
No correction in first 4 months	NIL	NIL	All joints studied have measurable correction after 4 months
			*p*-value = 1.0 (NS)
Physeal penetration	NIL	NIL	*p*-value = 1.0 (NS)
Implant migration/extrusion	Two knee joints (6.25 %)	NIL	*p*-value = 0.49 (NS)
Implant breakage	NIL	NIL	*p*-value = 1.0 (NS)
Failure of correction after 2 years	Two knee joints (6.3 %) both belonging to skeletal dysplasia (abnormal physes)	Two knee joints (6.5 %) both belonging to skeletal dysplasia (abnormal physes)	*p*-value = 1.0 (NS)
Requirement of additional procedure (osteotomy)	Four knee joints (12.5 %)	Two knee joints (6.5 %)	*p*-value = 0.43 (NS)
Overcorrection more than 5°	NIL	NIL	*p*-value = 1.0 (NS)
Recurrence	NIL	NIL	*p*-value = 1.0 (NS)
Limb length discrepancy more than 1 cm	Three cases, all unilateral (two idiopathic and one post-rickets genu valgum)	Two cases, both unilateral (both belonged to idiopathic genu valgum)	*p*-value = 1.0 (NS)
Total	7 (22 %)	4 (13 %)	*p*-value = 0.51 (NS)

*NS* not statistically significant difference, *SIG* statistically significant difference

## Discussion

Blount (1948) introduced staples as a means to achieve temporary hemiepiphysiodesis [4]. Although widely used and considered safe, several reports have documented the occurrence of complications with the use of staples [9, 11, 12]. Eight plate was introduced by Stevens as a means to avoid these complications [9]. Hemiepiphysiodesis is based on the Hueter–Volkmann principle, which states that compression and tension forces at the physis can cause physeal growth inhibition and acceleration, respectively [17]. Contrary to staples, which make a rigid construct providing compression to the physis throughout its length, eight plates act as a dynamic construct with scope for mobility at the screw–plate interface. This is the reason for staples having a higher risk of breakage and extrusion. In eight plate, maximum compression acts on the peripheral part of the physis at the screw plate junction, where it is actually required. Temporary hemiepiphysiodesis is applicable to skeletally immature children with open physes and having at least 1 year of growth remaining. Past studies had included a wide range of age groups, from as young as 2 years to those approaching skeletal maturity. In our study also, we had maintained this wide range (3.5–12 years in the staples group and 4–12 years in the eight plate group). To the best of our knowledge, only three previous studies have compared the results of staples with eight plate [14–16]. These are summarized in Table 4. In a retrospective study by Jelinek et al., during the period 1999–2008, a total of 33 knee angular deformities were treated with figure of eight plate and 32 with staples [14]. The rate of correction was similar in both treatment groups. They reported overcorrection in four cases of eight plate, which we avoided by close follow-up after correction was achieved. This is an avoidable complication. Migration of staples occurred in two cases, which is similar to our series.

**Table 4:** Summary of previous studies comparing the results of staples and eight plates application

	Wiemann et al. [15]	Jelinek et al. [14]	Gottliebsen et al. [16]	Our study
Number of knee joints studied	Staple = 39	Staple = 32	Staple = 10	Staple = 32
	Eight plate = 24	Eight plate = 33	Eight plate = 10	Eight plate = 31
Age distribution (in years)	Staple group = 12.6 (range: 8.5–16.7)	Staple group = 11.6 (range: 2.9–16)	Staple group = 11.1 (range: 6–13)	Staple group = 7.3 (range: 3.5–12)
	Eight plate group = 11.1 (range: 5.2–16)	Eight plate group = 13 (range: 10.5–15.2)	Eight plate group = 10.1 (range: 8–14)	Eight plate group = 7.8 (range: 4–12)
	*p* = 0.04 (NS)	*p* = 0.053 (NS)	*p* > 0.05 (SIG)	*p* = 0.53 (NS)
Male:female ratio	Staple group = 24:15	Staple group = 11:7	Staple group = 3:7	Staple group = 8:10
	Eight plate group = 11:13	Eight plate group = 8:9	Eight plate group = 8:2	Eight plate group = 8:11
	*p* = 0.298 (NS)	*p* = 0.50 (NS)	*p* < 0.05 (SIG)	*p* = 1 (NS)
Abnormal physes	Staple = 11	Staple = 5	NIL	Staple = 2
	Eight plate = 7	Eight plate = 4		Eight plate = 2
	*p* = 1.0 (NS)	*p* = 1.0 (NS)		*p* = 1.0 (NS)
Mean correction rate (° per month)	mLDFA = NA mMPTA = NA TFA = 0.825° for staples, 0.925° for eight plate *p* = 0.489 (NS)	mLDFA = 1° (range: 0–2) for both staples and eight plate *p* = 0.35 (NS)	mLDFA = NA mMPTA = NA TFA = NA	mLDFA = 1.0° (range: 0.75–1.25) for staples and 1.3° (range: 0.81–2.25) for eight plate *p* = 0.004 (SIG)
		mMPTA = 1° (range: 0–2) for both staples and eight plate *p* = 0.56 (NS)		mMPTA = 0.9° (range: 0.75–1.11) for staples and 0.8° (range: 0.56–1.12) for eight plate *p* = 0.001 (SIG)
		TFA = NA		TFA = 1.2° (range: 0.75–2.33) for staples and 1.4° (range: 1–2) for eight plate *p* = 0.70 (NS)
Mean time of implant removal	NA	Eight plate 11.9 ± 6.8 months (range: 1.9–27.9 months)	Eight plate 11.33 months (range: 9.5–13.1 months)	Eight plate 10.31 months
		Staple 11.0 ± 6.2 months (range: 4.5–28.1 months)	Staple 11.63 months (range: 8.76–14.5 months)	Staple 12.03 months
		*p* = 0.555 (NS)	*p* = 0.8 (NS)	*p* = 0.543 (NS)
Complications	Eight plate: Screw breakage = 1 Failure to correct = 3	Eight plate: Overcorrection = 4	Eight plate: Rebound growth = 1	Eight plate: Failure = 2 Limb length discrepancy >1 cm = 2
	Staples: Implant migration = 1 Failure to correct = 2 Overcorrection = 3	Staples: Post-operative wound site infection = 1 Implant migration = 2	Staples: None	Staples Failure = 2 Limb length discrepancy >1 cm = 3 Implant migration = 2
	*p* = 1.0 (NS) Limb length discrepancy = NA	*p* > 0.005 (NS) Failure to correct = NA		*p* = 0.51 (NS)

*NA* not mentioned in study, *NS* not statistically significant difference, *SIG* statistically significant difference, *mMPTA* mechanical medial proximal tibial angle, *mLDFA* mechanical lateral distal femoral angle, *TFA* anatomical tibio-femoral angle

In a retrospective study by Wiemann et al. [15], among 38 patients (24 extremities treated with eight plate and 39 with Blount staple temporary hemiepiphysiodesis), the rate of correction was higher in the eight plate group, but the difference was not statistically significant. Notably, they reported one case of eight plate screw breakage. The deformity correction rates in our study were comparable to past studies. The tibio-femoral angle (TFA) is a simple and reproducible measurable parameter on standing orthoroentgenogram. The overall correction rate of TFA in our study was 1.2° per month for the staple group and 1.4° per month for the eight plate group, the difference being statistically insignificant. The improvement in mMPTA was significantly lower in the eight plate group. This could be due to the small number of cases with deformity involving the proximal tibia. In skeletal dysplasia patients with abnormal physes, the correction rate was low in both groups. In idiopathic genu valgum, the correction rate was significantly lower in the staple group.

There were two knee joints with abnormal physis (skeletal dysplasia) in each group. Complications were similar to previous studies. The complication rate was slightly higher in the staple group, but this was statistically insignificant. There was no implant breakage in our series. Implant extrusion was seen in two joints in the staple group. Two failures (persistence of deformity at 2 years) were seen in both groups. Limb length discrepancy of more than 1 cm was seen in three cases in the staple group and two cases in the eight plate group. This has not been described in previous studies [14–16]. There was no case of sagittal plane deformity (flexion deformity or recurvatum deformity) in either group.

Early physiotherapy was recommended to ensure rapid return to daily activities and limit absence from school [18, 19].

Our study was not without limitations. There was an unequal distribution of varus and valgus knees, with valgus knees outnumbering varus knees in both groups. In our study, the minimum follow-up period was 2 years. Long-term follow-up studies for outcomes of eight plate are still needed. The patients with abnormal mMPTA were fewer than those with abnormal mLDFA. A larger number of the former could have helped in the better analysis of correction of mMPTA.

## Conclusion

Both staples and eight plates have similar potential for the correction of angular deformity around the knee in skeletally immature children. However, eight plates have a significantly higher rate of correction in idiopathic deformities. Allowing some overcorrection before implant removal (5° in our study) can prevent rebound deformity. Unlike eight plates, staples carry a risk of migration and extrusion. Abnormal physes as in skeletal dysplasias can lead to inferior outcomes in both groups.
